# Antihypertensive therapy responsiveness and adverse outcomes in preeclampsia: insights into molecular mechanisms underlying cardiovascular and renal complications

**DOI:** 10.3389/fphar.2023.1281382

**Published:** 2023-11-22

**Authors:** Marcelo R. Luizon, Daniela A. Pereira, Izabela Mamede, Carla S. Ceron, Ricardo C. Cavalli, Ana C. Palei, Valeria C. Sandrim

**Affiliations:** ^1^ Department of Genetics, Ecology and Evolution, Institute of Biological Sciences, Federal University of Minas Gerais, Belo Horizonte, Minas Gerais, Brazil; ^2^ Department of Biophysics and Pharmacology, Institute of Biosciences, Universidade Estadual Paulista (UNESP), Botucatu, São Paulo, Brazil; ^3^ Department of Biochemistry and Immunology, Institute of Biological Sciences, Federal University of Minas Gerais, Belo Horizonte, Minas Gerais, Brazil; ^4^ Department of Biological Sciences, Institute of Exact and Biological Sciences, Federal University of Ouro Preto, Ouro Preto, Minas Gerais, Brazil; ^5^ Department of Gynecology and Obstetrics, Ribeirao Preto Medical School, University of Sao Paulo, Ribeirao Preto, São Paulo, Brazil; ^6^ Department of Surgery, University of Mississippi Medical Center, Jackson, MS, United States

**Keywords:** antihypertensive agents, endothelial dysfunction, genetic polymorphisms, matrix metalloproteinase (MMP)-2, nicotinamide phosphoribosyltransferase (NAMPT), nitric oxide, preeclampsia, tissue inhibitor of metalloproteinase (TIMP)-3

## 1 Introduction

Preeclampsia (PE) is a multisystem disorder that affects 2%–8% of pregnancies worldwide and is a leading cause of maternal and fetal morbidity and mortality (Duley, 2009). PE is defined as new-onset maternal blood pressure greater than 140/90 mmHg after the 20th week of pregnancy that occurs along with proteinuria or other indications of renal insufficiency, thrombocytopenia, liver dysfunction, pulmonary edema, and cerebral disturbances (American College of Obstetricians and Gynecologists, 2013). The pathogenesis of PE is multifactorial with recognized placental, vascular, renal, and immunological contributions (Turbeville and Sasser, 2020).

PE is characterized by defective placentation, abnormal spiral artery remodeling, placental ischemia, oxidative stress at the maternal-fetal interface, and angiogenic imbalance in the maternal circulation, thereby resulting in endothelial dysfunction and end-organ damage (Phipps et al., 2019). Noteworthy, several studies ratify the relationship of PE with future risk for cardiovascular disease, and accumulating evidence suggests an association of PE with long-term renal disease, although further studies on the mechanisms underlying this increased risk are needed (Turbeville and Sasser, 2020). However, significant knowledge gaps still exist in identifying the mechanisms that link placental ischemia to maternal systemic vascular and renal dysfunction (Wang et al., 2023).

Current treatment strategies for PE focus on stabilizing the maternal symptoms in order to prolong pregnancy and allow additional fetal development, and managing maternal hypertension is a priority (Townsend et al., 2016; Wang et al., 2023). Antihypertensive drugs used to control high blood pressure, such as methyldopa, nifedipine, hydralazine, and labetalol have the potential to prolong gestation, decreasing obstetric and perinatal complications in PE (Berzan et al., 2014; Peracoli et al., 2019; American College of Obstetricians and Gynecologists, 2020). However, a large subgroup of patients with PE is nonresponsive to antihypertensive therapy, being more susceptible to develop adverse maternal and fetal outcomes (Luizon et al., 2017a).

In this opinion article, we contribute with viewpoints on the definition of phenotypes for the subgroup of patients with PE classified as nonresponsive to antihypertensive therapy, and how studies focused on this nonresponsive subgroup of patients with PE can yield insights into molecular mechanisms underlying the endothelial dysfunction as well as the associated cardiovascular and renal complications in PE.

## 2 Defining the phenotype: the subgroup nonresponsive to antihypertensive therapy in PE

Our group has previously used a criteria to define antihypertensive therapy responsiveness based on the evaluation of clinical and laboratory parameters following the administration of methyldopa, nifedipine and hydralazine (Sandrim et al., 2010a; Palei et al., 2012b; Luizon et al., 2014; Sandrim et al., 2015; Luizon et al., 2017a; Luizon et al., 2017b; Pereira et al., 2021). Notably, patients with PE classified as nonresponsive to antihypertensive therapy were markedly associated with the worst clinical parameters. However, many of the clinical findings we used on the definition of antihypertensive therapy responsiveness are shared with the definition of severe features in PE, including thrombocytopenia, abnormally elevated levels of liver enzymes and/or creatinine in the blood, persistent right upper quadrant or epigastric pain, and new-onset cerebral and visual disturbances (American College of Obstetricians and Gynecologists, 2013). Thus, it is possible that our criteria of responsiveness denote disease severity instead (Luizon et al., 2017a).

**FIGURE 1:**
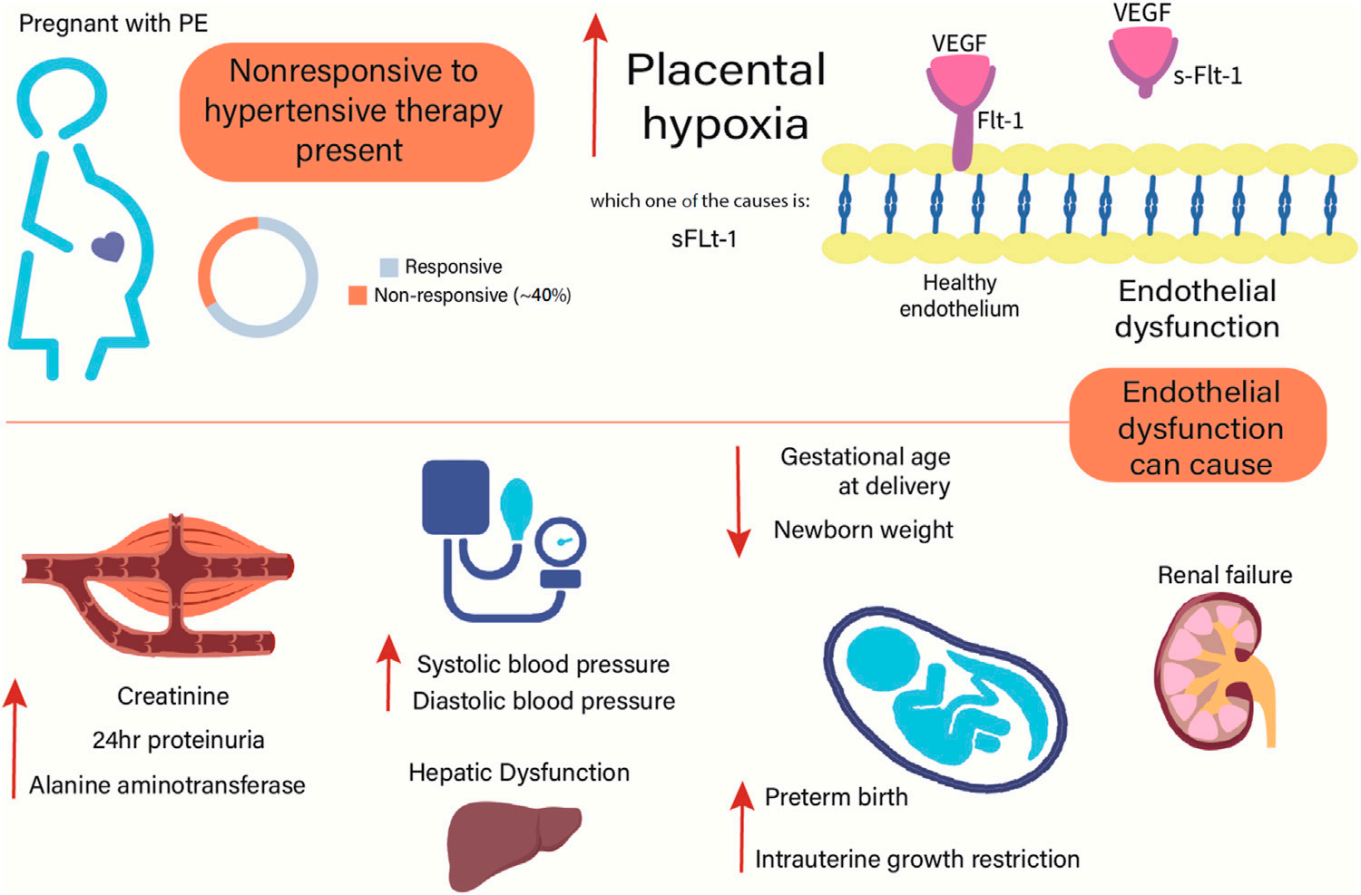
Clinical characteristics of patients with preeclampsia classified as nonresponsive to current antihypertensive therapy. Compared to the responsive group, the nonresponsive group showed higher systolic and diastolic blood pressure, creatinine, proteinuria, alanine aminotransferase, and sFlt-1 levels (Pereira et al., 2021). Adverse outcomes in the mother and fetal development may be implicated to the endothelial dysfunction observed in PE. Conversely, the nonresponsive group showed lower gestational age at delivery and newborn weight than the responsive group (Pereira et al., 2021). sFlt-1, soluble fms-like tyrosine kinase-1; VEGF, vascular endothelial growth factor.

Noteworthy, we have recently better characterized the clinical phenotype of patients with PE classified as nonresponsive to antihypertensive therapy (Pereira et al., 2021). The nonresponsive subgroup showed higher blood pressure, fasting glucose, creatinine, proteinuria, alanine aminotransferase, and soluble fms-like tyrosine kinase-1 (sFlt-1) levels, in opposition to lower gestational age at delivery and newborn weight (Figure 1). Other symptoms related to impairment of the central nervous system, blood cells (hemolysis and thrombocytopenia), and fetal development were also recorded (Pereira et al., 2021). Furthermore, PE can be classified according to gestational age of onset of symptoms into early-onset PE (<34 weeks) and late-onset PE (≥34 weeks). Remarkably, the subgroup nonresponsive to antihypertensive therapy showed higher percentage of early-onset PE (47.3% versus 6.3%), preterm birth (61.5% versus 11.6%), and intrauterine growth restriction (50.5% versus 14.3%) than the responsive subgroup (Pereira et al., 2021).

Although a large subgroup (∼46%) of patients with PE are nonresponsive to currently approved antihypertensive therapy during pregnancy (Luizon et al., 2017a), the underlying molecular mechanisms are unclear. In the next sessions, we contribute with viewpoints on the interpretation of findings from previous studies focused on this nonresponsive subgroup of patients with PE and how they can yield insights into the underlying mechanisms of increased cardiovascular and renal risk in PE.

## 3 Visfatin/extracellular nicotinamide phosphoribosyltransferase (eNAMPT): nitric oxide (NO) bioavailability and endothelial dysfunction

Antiangiogenic factors released into the maternal circulation, including sFlt-1 as result of placental ischemia/hypoxia contribute to the widespread endothelial dysfunction and proteinuria found in PE (Maynard et al., 2003; Powe et al., 2011). Circulating nitrite concentrations (a marker of endogenous NO formation) is a suitable approach to understand the underlying molecular mechanisms of endothelial dysfunction in healthy subjects (Metzger et al., 2013), healthy pregnancy and in PE (Sandrim et al., 2010b), as reviewed elsewhere (Luizon et al., 2018). Notably, PE is characterized by reduced bioavailability of NO, which is inversely related to sFlt-1 (Sandrim et al., 2008).

Endothelial dysfunction in PE has also been associated with dysregulation of adipocytokines (Mori et al., 2010). Specifically, visfatin/eNAMPT is an adipocytokine that has been proposed as a marker of endothelial dysfunction and vascular damage (Romacho et al., 2013), and visfatin/eNAMPT was shown to produce *in vivo* endothelial dysfunction in mice via toll-like receptor-4 (TLR4)-mediated pathway (Romacho et al., 2020). Potential interactions among visfatin/eNAMPT, TLR4, and inflammatory cytokines in PE should be further considered (Nunes et al., 2022). Briefly, possible visfatin/eNAMPT mechanisms of action by binding to TLR4 activates NF-kB phosphorylation and its translocation to the nucleus, where it acts by activating the NLRP3 inflammasome, responsible for the release of inflammatory cytokines. Activation of NLRP3 also releases reactive oxygen species, increasing oxidative stress, and therefore inhibiting NO formation, as reviewed elsewhere (Nunes et al., 2022).

Most studies provided evidence for increased visfatin levels in pregnant women with PE, despite discordant findings (Amiri-Dashatan et al., 2022). Although we have found no differences in the circulating levels of visfatin/eNAMPT between health pregnancy and PE (Luizon et al., 2015), visfatin/eNAMPT levels were inversely related to circulating NO and positively related to sFlt-1 levels in PE (Pereira et al., 2019). Notably, the same correlations were significantly only in the subgroup of patients with PE classified as nonresponsive to antihypertensive therapy, who showed higher proteinuria and plasma sFlt-1 levels (Pereira et al., 2021).

Visfatin impairs endothelium-dependent relaxation through nicotinamide adenine dinucleotide phosphate hydrogen (NADPH) oxidase activity, which leads to release of superoxide anions (Vallejo et al., 2011). These anions scavenge NO to generate peroxynitrite, which stimulates the expression of inducible NO synthase enzyme and intercellular cell adhesion molecule-1, a well-known marker of endothelial dysfunction (Goulopoulou and Davidge, 2015). Superoxide anions also induce the uncoupling of endothelial NO synthase (Brennan et al., 2014), further decreasing NO bioavailability. During pregnancy, an increase in oxidative stress is observed as a result of the normal systemic inflammatory response, reflected as higher levels of circulating reactive oxygen species. This increased oxidative stress does not cause tissue damage because it is counter-balanced by the synthesis of antioxidants (Chiarello et al., 2020). However, in PE, placental ischemia/hypoxia exacerbates increased oxidative stress to a level that is harmful for the mother and fetal health by decreasing NO bioavailability as well as via several other mechanisms (Chiarello et al., 2020; Guerby et al., 2021).

Taken together, the evidence provided in the preceding paragraphs suggests that visfatin/eNAMPT may inhibit NO formation and upregulate sFlt-1, which may be due to the increased oxidative stress observed in PE. In view of the complex deleterious impact that adipocytokines have on the endothelium and vascular homeostasis (McElwain et al., 2020), these potential mechanisms may underly the role of visfatin/eNAMPT in vascular dysfunction in PE, as reviewed elsewhere (Ceron et al., 2022; Ceron et al., 2023), and perhaps serve as a link between PE and the occurrence of cardiovascular and renal diseases later in life.

## 4 Matrix metalloproteinase (MMP)-2 and tissue inhibitor of metalloproteinase (TIMP)-3: insights into epigenetic mechanisms of endothelial dysfunction

MMP-2 is expressed by a variety of cells including trophoblasts, endothelial cells, and fibroblasts, playing a major role in embryogenesis, placental morphogenesis, and cardiovascular and renal function (Palei et al., 2013). We have found higher circulating MMP-2 levels in patients with PE classified as nonresponsive to antihypertensive therapy (Palei et al., 2012a).

In addition, we examined the differential gene expression in human umbilical vein endothelial cells (HUVECs) incubated with plasma from patients with PE classified according to antihypertensive therapy (nonresponsive relative to responsive patients), and we identified interactions among genes and antihypertensive drugs used in PE (Luizon et al., 2016). Notably, genes that were downregulated or upregulated in HUVECs incubated with plasma from nonresponsive PE patients were reported as upregulated or downregulated by nifedipine and hydralazine, respectively. Notably, while *MMP-2* was found to be upregulated in these HUVECs (Luizon et al., 2016), hydralazine treatment was shown to decrease *MMP-2* expression in spontaneously hypertensive rats (Kodavanti et al., 2013).

Hypoxia can stimulate *MMP-2* expression, and MMP-2 is capable of cleaving big endothelin (ET)-1 and induce hypertension through the generation of ET-1, a potent vasoconstrictor (Fernandez-Patron et al., 1999). Interestingly, we found that plasma from patients with PE stimulated microRNA expression in HUVECs that were negatively related to ET-1 levels (Caldeira-Dias et al., 2018). Considering that agents that improve endothelial function in PE hold promise to alleviate clinical symptoms (Sasser et al., 2015), microRNAs may be useful candidates for therapeutic intervention in the management of hypertension in PE (Caldeira-Dias et al., 2018).

As aforementioned, patients with PE classified as nonresponsive to antihypertensive therapy showed higher plasma creatinine and proteinuria (Figure 1) than responsive patients (Pereira et al., 2021). Creatinine is considered a fairly reliable indicator of kidney function. Proteinuria is also associated with impaired renal function in PE, being the amount of protein loss related to disease severity (Fishel Bartal et al., 2022). Patients with PE have angiogenic imbalance represented by increased circulating sFlt-1 and soluble endoglin, but reduced vascular endothelial growth factor (VEGF) and placental growth factor levels, which together damage the endotelium of the glomerular filtration barrier (Fishel Bartal et al., 2022). In this regard, clinical and experimental studies reviewed elsewhere (Cruz et al., 2021) have shown a compelling role for the full-length MMP-2 in ischemic renal injury, progressive renal fibrosis, and diabetic nephropathy. Interestingly, evidence suggests that MMP-2 is able to cleave the extracellular domain of the VEGF receptor-2, thereby leading to endothelial apoptosis and vascular rarefaction (Tran et al., 2010; Wang et al., 2014).

While the extracellular activity of MMP-2 is mainly regulated via inhibition by TIMPs, a novel intracellular N-terminal truncated isoform of MMP-2 has been discovered, which is induced by hypoxia and oxidative stress by activation of a latent promoter located in the first intron of *MMP-2* (Lovett et al., 2012). We have previously proposed that the latent promoter of this MMP-2 isoform undergoes epigenetic regulation via its overlap with histone modifications, a putative active enhancer element, and binding sites for transcription factors that are known to cooperate in hypoxia-induced gene transcription (Cruz et al., 2021).

The balance between MMPs and TIMPs is key to the stability of the extracellular matrix and normal function of these proteases during pregnancy. Although TIMP-3 is able to bind and inhibit MMP-2, it has also been reported that activation of the pro-form of MMP-2 by Matrix type 3-MMP may be enhanced by TIMP-3 in a dose-dependent manner (Zhao et al., 2004). Furthermore, TIMP-3 has MMP-independent functions, such as the inhibition of angiogenesis by blocking the binding of VEGF to VEGF receptor-2 and hindering downstream signaling (Qi et al., 2003). Regarding the role of *TIMP-3* in PE, the promoter polymorphism rs9619311 was not associated with response to antihypertensive therapy (Luizon et al., 2014). However, we identified a higher number of significant differentially methylated probes located on the *TIMP-3* promoter, as well as an increased *TIMP-3* expression in corresponding placental samples from early-onset PE compared to controls, which denotes DNA methylation of *TIMP-3* promoter as an epigenetic mechanism in PE (Cruz et al., 2022). Recently, we found that circulating TIMP-3 is increased in patients with PE compared with healthy pregnancy, and these TIMP-3 levels were positively correlated with MMP-2 and TIMP-1 concentrations in PE (Palei et al., 2022). These findings may contribute to understand the relevance of TIMP-3 in the pathophysiology of PE.

## 5 Conclusion

Here, we discussed potential mechanisms underlying endothelial dysfunction and associated long-term cardiovascular and renal risk in PE based on findings from studies focused on patients with PE classified as nonresponsive to antihypertensive therapy. Effects on NO bioavailability highlight the potential role of visfatin/NAMPT on mediating endothelial dysfunction, which might contribute to increased cardiovascular events in PE. Moreover, we provided insights into the epigenetic control of *MMP-2* and *TIMP-3* expression that may contribute to cardiovascular and renal complications in PE. Finally, epigenetic regulation of the vascular endothelium should be further considered as potential drug targets to improve antihypertensive responsiveness in PE.
